# Monitoring iCCM referral systems: Bugoye Integrated Community Case Management Initiative (BIMI) in Uganda

**DOI:** 10.1186/s12936-016-1300-z

**Published:** 2016-04-29

**Authors:** Lacey English, James S. Miller, Rapheal Mbusa, Michael Matte, Jessica Kenney, Shem Bwambale, Moses Ntaro, Palka Patel, Edgar Mulogo, Geren S. Stone

**Affiliations:** School of Medicine, University of North Carolina at Chapel Hill, Chapel Hill, NC USA; Harvard Medical School, Boston, MA USA; Bugoye Health Centre III, Bugoye, Uganda; Department of Community Health, Mbarara University of Science and Technology, Mbarara, Uganda; Global Primary Care Program, Massachusetts General Hospital, Boston, MA USA

**Keywords:** Africa, Child mortality, Case management, Referral, Paediatric malaria

## Abstract

**Background:**

In Uganda, over half of under-five child mortality is attributed to three infectious diseases: malaria, pneumonia and diarrhoea. Integrated community case management (iCCM) trains village health workers (VHWs) to provide in-home diagnosis and treatment of these common childhood illnesses. For severely ill children, iCCM relies on a functioning referral system to ensure timely treatment at a health facility. However, referral completion rates vary widely among iCCM programmes and are difficult to monitor. The Bugoye Integrated Community Case Management Initiative (BIMI) is an iCCM programme operating in Bugoye sub-county, Uganda. This case study describes BIMI’s experience with monitoring referral completion at Bugoye Health Centre III (BHC), and outlines improvements to be made within iCCM referral systems.

**Methods:**

This study triangulated multiple data sources to evaluate the strengths and gaps in the BIMI referral system. Three quantitative data sources were reviewed: (1) VHW report of referred patients, (2) referral forms found at BHC, and (3) BHC patient records. These data sources were collated and triangulated from January–December 2014. The goal was to determine if patients were completing their referrals and if referrals were adequately documented using routine data sources.

**Results:**

From January–December 2014, there were 268 patients referred to BHC, as documented by VHWs. However, only 52 of these patients had referral forms stored at BHC. Of the 52 referral forms found, 22 of these patients were also found in BHC register books recorded by clinic staff. Thus, the study found a mismatch between VHW reports of patient referrals and the referral visits documented at BHC. This discrepancy may indicate several gaps: (1) referred patients may not be completing their referral, (2) referral forms may be getting lost at BHC, and, (3) referred patients may be going to other health facilities or drug shops, rather than BHC, for their referral.

**Conclusions:**

This study demonstrates the challenges of effectively monitoring iCCM referral completion, given identified limitations such as discordant data sources, incomplete record keeping and lack of unique identifiers. There is a need to innovate and improve the ways by which referral compliance is monitored using routine data, in order to improve the percentage of referrals completed. Through research and field experience, this study proposes programmatic and technological solutions to rectify these gaps within iCCM programmes facing similar challenges. With improved monitoring, VHWs will be empowered to increase referral completion, allowing critically ill children to access needed health services.

**Electronic supplementary material:**

The online version of this article (doi:10.1186/s12936-016-1300-z) contains supplementary material, which is available to authorized users.

## Background

Each year approximately 3.3 million deaths in children under 5 years old occur due to infectious diseases [1]. In Uganda, the child mortality rate is 6.9 %, with malaria, diarrhoea and pneumonia specifically accounting for over half of these child deaths [2]. Often these deaths are due to delays in recognizing an illness, deciding to seek care, or reaching the health facility [3–6]. Integrated community case management (iCCM) was developed to address these delays, improve access to treatment of common childhood illnesses, and thereby decrease child mortality [7].

The iCCM strategy trains village health workers (VHWs) to visit sick children under 5 years old and provide in-home diagnosis and treatment of malaria, pneumonia and diarrhoea in communities with limited health infrastructure [8]. An essential component of iCCM is a functioning referral system to ensure children with more severe illnesses, who fall outside the VHW scope of practice, receive timely medical care at a health facility. However, studies have shown iCCM referral completion rates to be quite varied, ranging from 1.5 to 87 % [9–11]. Low completion rates are concerning as they may indicate high-risk children are not receiving critically needed care.

The Bugoye Integrated Community Case Management Initiative (BIMI), an iCCM programme operating in Bugoye Sub-County, Uganda, collects routine data to evaluate the referral system component of their programme. The BIMI programme is built upon collaboration between the Mbarara University of Science and Technology (MUST), the MGH Global Primary Care (GPC) Programme, and Bugoye Health Centre III (BHC). This report describes BIMI’s efforts to understand referral completion, and the important yet difficult nature of monitoring VHW referral systems in resource-limited settings. Through retrospective review, 2014 BIMI data are presented to reflect upon demonstrated challenges to using routine data, as well as to propose future solutions given lessons learned.

### Overview of BIMI

The BIMI programme enlists, trains and supplies 23 VHWs to provide iCCM services within five villages surrounding BHC: Bugoye, Ihani, Kanyminigo, Kikokera, and Muramba, in the foothills of the Rwenzori mountains in western Uganda. These five villages were chosen by the local government and BHC leadership with the goal of covering the most people presenting to BHC with malaria. While these villages may not truly encompass the highest prevalence rates, they represented the areas where malaria places the highest burden on the local health care system. Bugoye sub-county is home to approximately 36,000 people with an average household size of 5.6 people. Malaria and respiratory tract infections are the leading illnesses in the district [12].

The VHW team was elected by its village community, with four to five VHWs derived from each of the five aforementioned villages. They received thorough training to appropriately diagnose, treat and/or refer children under five years old with malaria, pneumonia or diarrhoea. Initially, VHWs participated in a basic 3-day training standardized by the Ugandan Ministry of Health (MoH) to review VHW responsibilities. This was followed with a 6-day training on implementing iCCM services. To supplement the foundational training, VHWs receive quarterly refresher training at BHC.

A standardized job aid, issued by the Ugandan MoH, was used to train the VHW team to diagnose, treat and refer ill children in the community (Additional file 1). Given the high prevalence, malaria rapid diagnostic tests (RDT) are given to every sick child. The job aid guides VHWs through the following steps listed in Table 1. Referral is indicated when a patient exhibits the following danger signs: (1) prolonged symptoms, (2) newborn with skin pustules or infected umbilical cord, or, (3) severe symptoms such as convulsions, chest indrawing, vomiting, anorexia, or unconsciousness. With recognition of danger sign(s), VHWs give pre-referral treatment to initiate care or attenuate symptoms during the time to reach the nearest health centre. If the child has experienced diarrhoea for 14 or more days, the VHW gives pre-referral oral rehydration solution (ORS). If the child has experienced fever for seven or more days, the VHW gives the first dose of anti-malarial artemisinin-based combination therapy (ACT). If the child experiences fever plus a danger sign, the VHW gives rectal artesunate to pre-emptively treat for severe malaria, but if the child experiences chest indrawing plus a danger sign, the VHW gives amoxicillin to pre-emptively treat for severe pneumonia [13].

**Table 1:** Summary of Ugandan MoH job aid for VHW iCCM training

Steps in the job aid	Indicated actions
Step 1: Ask caregiver for child’s age	Mark age up to 5 years old
Step 2: Ask caregiver about child’s symptoms	Mark cough, diarrhoea and/or fever If cough present, check for fast breathing
Step 3: Ask caregiver and look for danger signs	If any danger signs present, refer to local health centre
Step 4a: Pre-referral treatment if danger signs	Give ORS, amoxicillin, ACT, or rectal artesunate depending on the danger sign(s) present
Step 4b: Treat and advise if no danger signs	For cough, give amoxicillin For diarrhoea, give ORS and zinc For fever, give ACT
Step 5: Advise for all children treated at home	Give more fluids Go to health centre if notice danger signs Sleep under bed net Follow up with VHW in 3 days
Step 6: Advise on routine care of newborns	Keep baby warm Exclusive breastfeeding Skin and cord cleanliness

### Overview of the BIMI referral system

**Fig. 1:**
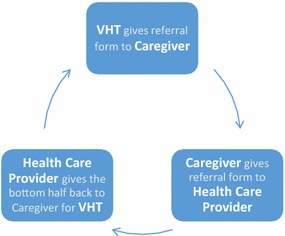
Overview of the BIMI referral system

Since beginning in March 2013, VHWs have provided over 6300 treatments for malaria, pneumonia and/or diarrhoea to children in their community, as well as referred 560 patient to BHC for additional care. The BIMI programme structures its referral system in accordance with the Ugandan MoH job aid and iCCM guidelines [14]. When a child is referred, the VHW provides the caregiver with a referral form and instructs the family to visit BHC, or accompanies the family there. At the health centre, the healthcare provider retains the top half of the referral form but sends the bottom half back with the caregiver for the VHW. On the half of the form returned to the caregiver, the provider gives feedback on additional ways the VHW can support patient care at home. This system provides a feedback loop to ensure the patient attended their referral and received follow-up care (Fig. 1).

## Methods

This retrospective study aimed to assess the BIMI iCCM referral system, an integral component of improving child access to health care. Multiple data sources were triangulated to understand the strengths and gaps in the current referral system. This evaluation aimed to: (1) describe the iCCM referral system and, (2) ascertain the degree of patient compliance with referrals (Table 2).

**Table 2:** Summary of study aims

Evaluation aim	Quantitative indicator	Data source
Describe the iCCM referral system	Total number of children referred	Monthly reports
	Number and location of referral forms found at BHC	Referral forms
Ascertain the degree of patient compliance with referrals	Number of referral forms found at BHC	Referral forms
	Number of referred patients found in the 2014 BHC registers	2014 sick patient registers; 2014 IPD and OPD registers

This work was approved by the institutional review boards (IRB) at Mbarara University of Science and Technology in Mbarara, Uganda (Ref No. 04/11-11) and the Partners Institutional Review Board in Boston, MA, USA (Protocol No. 2012P000069). The BIMI iCCM programme operates in accordance with the standards of care prescribed by the Ugandan MoH. Per MoH guidelines, BIMI collects routine data for programmatic and clinical purposes. This routine data is approved for research purposes by the aforementioned IRBs. All patient data are de-identified and any personal identifiers are removed. Since BIMI operates within the prescribed standards of care for all patients who present for treatment and protect personal health information, the study did not seek additional consent from caregivers for retrospective review of routine data sources.

### Data sources and analysis

This study triangulated multiple data sources to evaluate the strengths and gaps in the BIMI referral system. Three quantitative data sources were reviewed: (1) patients indicated as referred by VHWs in the sick patient registers, (2) referral forms found at BHC and, (3) referred patients recorded in the BHC patient registers by clinic staff. The goal of reviewing these data sources was to determine if patients are completing their referrals and if referrals are adequately documented.

The sick patient registers are completed monthly by each VHW and summarize all iCCM patient encounters. In the register, the VHWs document the name and health conditions for each referred child. This study compiled the sick patient registers from January–December 2014 to determine the total number of children referred by VHWs.

The referral form is provided by the VHW to the patient at the time of referral, and proceeds with the patient through the referral system. Part of the form should be stored at the health centre to document patient visits due to VHW referral. This study reviewed the referral forms found at BHC from January–December 2014 to assess the number of referrals being completed by patients.

This study used BHC clinical registers to supplement referral form data for 2014 referrals, given the possibility of lost referral forms by the caregiver or health centre. BHC documents the name, age, sex, and village of patients seen at the inpatient department (IPD) and outpatient department (OPD) in register books. These register books should record all children who were seen at the health centre. To assess the number of patients completing referrals, this study reviewed the sick patient registers from January–December 2014 and compiled the name, age, sex, and village for all referred patients. These referred patients were then cross-checked in the 2014 IPD and OPD registers to confirm completion of their referrals, with or without referral forms.

The study defined set criteria for a ‘definite match’ and a ‘probable match’ between the sick patient register and BHC register, given the lack of a reliable unique identifier (e.g., date of birth, medical record number), as well as the potential for incomplete or inaccurate documentation. A definite match occurred when the patient’s full name, age and village were matching in both the sick patient register and BHC register. A probable match occurred when a referred patient’s full name and village from the sick patient register matched a patient’s information in the BHC register, but the age from these sources differed by ± one month for children under one year of age. For example, the referred patient is listed as seven months in the sick patient register, but a matching patient is listed as six months in the BHC register. Alternatively, a probable match also occurred when a referred patient’s age and village from the sick patient register matched a patient’s information in the BHC register, but their name was incompletely recorded. For example, the sick patient register records the child’s name as ‘Gift’, and a seemingly matching patient is listed as ‘Gift Niwe’ in the BHC register. For both definite and probable matches, the VHW visit with the referred patient (as recorded in the sick patient register) had to be within 1 month of the visit date found in the BHC registers. Outside of this time period, it was presumed the BHC visit was unlikely due to the VHW referral. For the purposes of this study, definite and probable matches were summed to estimate the referred patients found in the IPD and OPD registers.

## Results

### 2014 referral system in Bugoye

**Fig. 2:**
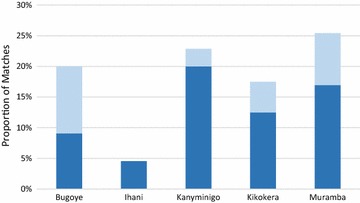
Matches between referral forms and BHC registers

**Fig. 3:**
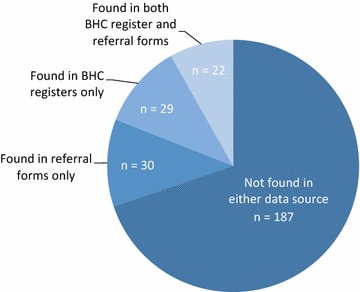
Concordance of BHC records among the 268 patients referred in 2014

From January–December 2014, there were 268 patients referred to BHC, as indicated in the sick patient registers. Of these patients, only 52 referral forms were found at BHC.^1^ This study found a mismatch between VHW reports of patient referrals and the number of referral forms found at BHC. Of the 52 referral forms found, 22 of these patients were also found in the IPD or OPD registers. The majority were definite matches between the two data sources, while approximately one-third were probable matches (Fig. 2). Among villages, the proportion of referral forms matching the BHC registers ranged from 4.5 % for Ihani to 25.4 % for Muramba. There were 30 patients whose attendance at BHC was confirmed by a referral form at the health centre, but whom were not listed in the IPD or OPD registers. An additional 29 referred patients were found in the IPD or OPD registers, but for whom a referral form stored at the health centre was not found (Fig. 3). Therefore, referral completion was estimated by adding the number of referral forms found at BHC (52 forms) with the number of referred patients found in the BHC register without a referral form filed at the health centre (29 patients). Overall, 81 patients were found in the BHC registers and/or had a referral form filed at the health centre to confirm their completion of the referral. Utilizing multiple sources of data, only 30 % of the 268 patients who were referred in 2014 were confirmed as completing their referral.

The discrepancy between the number of patients referred and the number of referral forms found may indicate several gaps: (1) referred patients may not be completing their referral; (2) referral forms may be getting lost at BHC; and, (3) referred patients may be going to other health facilities or drug shops, rather than BHC, for their referral.

It is possible patients completed their referral, but the form was left at home or misplaced at BHC. However, this would not fully explain the mismatch between number of patients referred and the number of referral forms found. This explanation does not account for the 30 patients for whom a referral form was found at BHC, but the patients’ visits were not documented in the BHC registers. There are several possible reasons for this discrepancy: (1) patients who were in fact recorded in the BHC registers could not be matched to the listing in the sick patient registers, due to the lack of unique identifiers, incomplete or incorrect information recorded on the sick patient registers, or incomplete or incorrect information recorded in the BHC register; (2) some patients were seen at BHC but left without being recorded in the BHC registers. Regardless, the discrepancy between these data sources highlights the challenges of using routine data, and leads to significant uncertainty regarding the completion or non-completion of referrals.

## Discussion

In this study, the confirmed referral completion rate (30 %) was low, even after investigating multiple data sources. The discrepancies found between data sources demonstrate the challenges of using routine data to monitor iCCM referral completion. Of note, some referral forms were found at BHC which did not match the BHC clinical registers, indicating health centre records may not be reflective of all patient visits. On the other hand, some children were documented as referred in the sick patient registers and found in the BHC registers, but without a referral form stored at BHC. This indicates routine data collection is not consistently capturing completed referrals by iCCM patients. To rectify these identified gaps, there is a need to innovate and improve the ways by which referral compliance is monitored, in order to improve the percentage of referrals completed.

Few strategies exist for monitoring referral completion. Some studies have used routine programmatic data, collected regularly by a health facility or VHWs, to monitor the iCCM referral system [9, 15]. The benefit of this approach is timely feedback on programmatic performance, but often these records are inaccurate or incomplete [9, 16, 17]. Independent evaluations, such as focus groups or self-report from caregivers, are an alternative method [3, 4, 18]. These provide greater depth of information, but increase the risk of selection and recall bias with only a small number of cases represented. Triangulation of data should be considered to counter weaknesses of any single source and improve iCCM referral monitoring.

Monitoring and evaluation (M&E) of iCCM referral systems is essential to ensuring severely ill children are completing referrals and receiving potentially life-saving treatment. Successful M&E can improve the efficiency of iCCM programmes and inform programmatic decision making [19]. However, this study demonstrates the challenges of effectively monitoring iCCM referral completion, given common limitations such as discordant data sources, incomplete record keeping and lack of unique identifiers. Few studies have been conducted on iCCM referrals, but some have documented similar difficulties [9, 17].

Similar to other iCCM programmes, the BIMI referral system requires coordination by multiple parties: VHWs, patients and BHC healthcare workers. In this case, the involvement of multiple players complicated tracking of misplaced data and limited the ability to pinpoint or quantify the exact reasons for missing data. Vulnerabilities with routine data collection included multiple steps in the referral process:

- Referred patients may not bring the referral form to BHC;
- Referred patients may go to another health facility or drug shop for their referral;
- Referral forms may be misplaced at BHC;
- Patient names may not be recorded in the BHC registers.

It is plausible that a caregiver may have thought their child’s illness not to be severe and therefore not completed the referral. Some danger signs indicating referral by the iCCM job aid, such as vomiting, can be caused by viral infections which tend to resolve on their own. Previous research has shown caregivers often do not recognize symptoms and danger signs of severely ill children, which may have delayed or dissuaded referral completion [20, 21]. Further research is needed to understand the context surrounding referral non-compliance.

## Conclusions

As exemplified by 2014 referral data, M&E improvements could be made among iCCM programmes to enable better monitoring of referral systems and patient referral outcomes in the future. Table 3 discusses key challenges observed through the BIMI case study and proposes solutions to consider for iCCM programmes facing similar challenges.

**Table 3:** Proposed solutions for challenges with routine data

Identified M&E challenges	Proposed solutions
Inadequate tracking of referral forms	Standard filing system for referral forms agreed upon by health centre staff Weekly collection of referral forms by staff to further ensure secure storage Periodic trainings with health centre staff and VHWs to review referral and data collection protocols Mobile health applications [22] including text message reminders or real-time documentation of referral placement and completion via mobile devices
Discordance between multiple data sources	Unique patient identifiers to simplify monitoring across data sources [23] Continued triangulation of multiple data sources, as seen in this study among others [8]
Inconsistent monitoring and evaluation of the referral system	Referral indicators in the monthly M&E report Monthly dashboard to compare multiple data sources Community-based quality improvement approaches, whereby health care workers receive regular feedback from M&E data collection and are actively involved in subsequent programmatic decision making [24]

Referral compliance is a multifaceted health behaviour, with social, educational and financial constraints often contributing to low compliance rates [4, 18]. However, few iCCM studies exist on interventions to overcome these barriers and improve referral completion rates. Strengthening routine data management would create opportunities to understand, test and intervene upon barriers to compliance. In addition, real-time monitoring of referrals would enable VHWs to follow-up with patients to ensure the health services are accessed. This active monitoring of referrals would encourage continuous systematic improvements, leading to more efficient and effective care for children in Bugoye, Uganda and other iCCM programmes.

## Additional file

10.1186/s12936-016-1300-z Sick child job aid. Ministry of Health, Uganda.

## Footnotes

1 Though not included in the study sampling frame, 14 referral forms were found for patients not listed as referred in the sick patient register, resulting in 66 referral forms found overall.
