# Erratum to: Monitoring iCCM referral systems: Bugoye Integrated Community Case Management Initiative (BIMI) in Uganda

**DOI:** 10.1186/s12936-016-1375-6

**Published:** 2016-07-04

**Authors:** Lacey English, James S. Miller, Rapheal Mbusa, Michael Matte, Jessica Kenney, Shem Bwambale, Moses Ntaro, Palka Patel, Edgar Mulogo, Geren S. Stone

**Affiliations:** School of Medicine, University of North Carolina at Chapel Hill, Chapel Hill, NC USA; Harvard Medical School, Boston, MA USA; Bugoye Health Centre III, Bugoye, Uganda; Department of Community Health, Mbarara University of Science and Technology, Mbarara, Uganda; Global Primary Care Program, Massachusetts General Hospital, Boston, MA USA

## Erratum to: Malar J (2016) 15:247 DOI 10.1186/s12936-016-1300-z

After publication of the original article [1], it came to the authors’ attention that a sentence was inadvertently omitted from the Acknowledgments section of the published manuscript. The section should have read as follows:

“Special thanks to Tinka Tadeo and his team of VHWs in Bugoye Sub-County for their assistance with this study, as well as their inspiring commitment to serving their community. We also want to thank Dr. Raquel Reyes for her leadership and service in the BIMI Program as the MGH Global Primary Care Uganda Site Director.”
