# Supplementary material for: Monitoring iCCM referral systems: Bugoye Integrated Community Case Management Initiative (BIMI) in Uganda
Source: Malar J. 2016 Apr 29;15:247. doi: 10.1186/s12936-016-1300-z (PMC4850682; doi:10.1186/s12936-016-1300-z)
Supplement: Supplementary file 1 — 10.1186/s12936-016-1300-z Sick child job aid. Ministry of Health, Uganda. [file 12936_2016_1300_MOESM1_ESM.pdf]

1. ASK THE CAREGIVER

How old is the child?

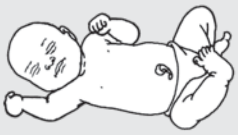

☐ **0 - 7 days**

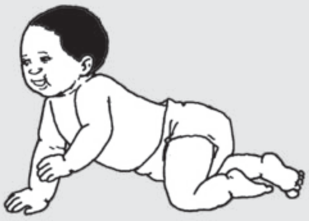

☐ **2 - 11 months**

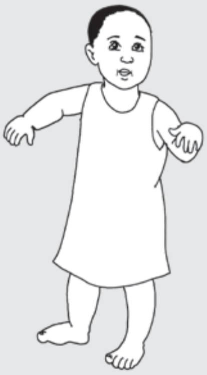

☐ **1 - 2 years**

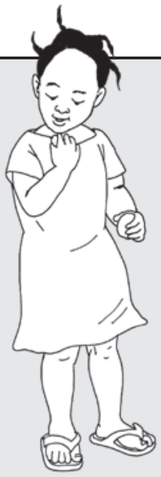

☐ **3 - 5 years**

2. ASK - CHILD'S PROBLEMS

Does the child have cough, diarrhoea or fever?

If yes, for how long?

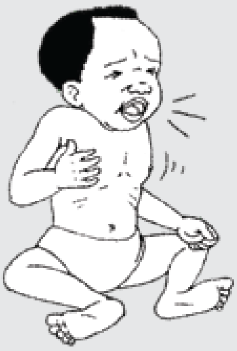

☐ **Cough**

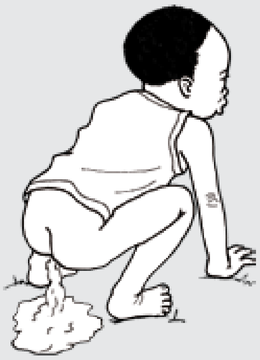

☐ **Diarrhoea**

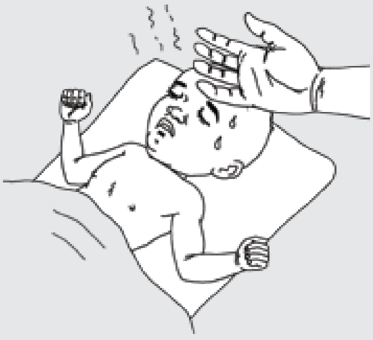

☐ **Fever**

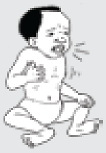

If **Cough** is present, check for **Fast Breathing**.

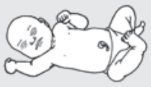

0 – 7 days

60 or more
breaths per minute

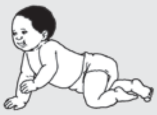

2-11 months

50 or more
breaths per minute

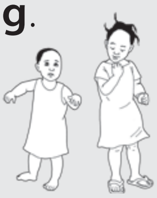

1-5 years

40 or more
breaths per minute

3. ASK AND LOOK FOR DANGER SIGNS AND REFER

Refer to Step 4a because children with some of these danger signs need pre-referral treatment.

Any child or newborn with...

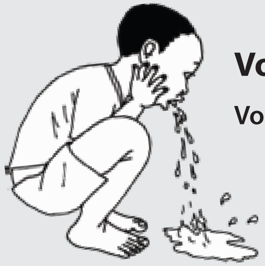

Vomiting ☐

Vomits everything

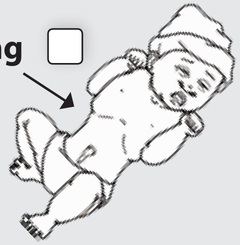

Chest in-drawing ☐

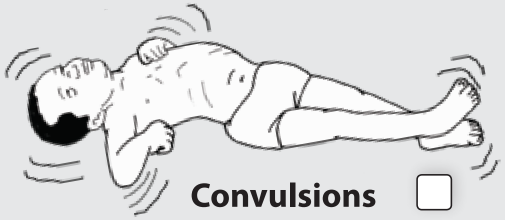

Convulsions ☐

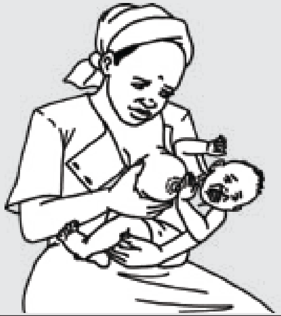

Not able to breastfeed or drink ☐

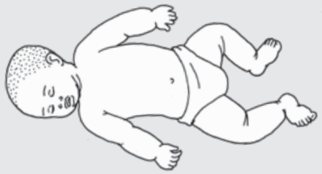

Very sleepy or unconscious ☐

A newborn with...

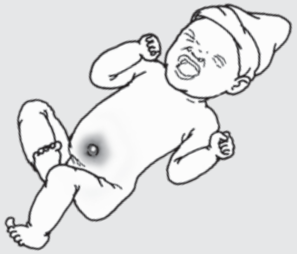

Infected umbilical cord ☐

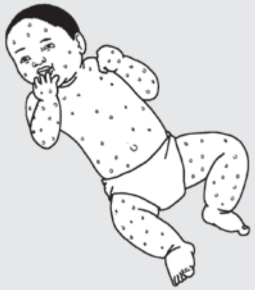

Many skin pustules ☐

A child with...

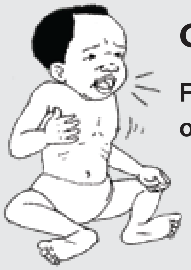

Cough ☐

For 21 days or more

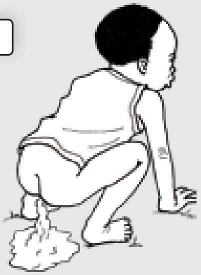

Diarrhoea ☐

For 14 days or more, or with blood

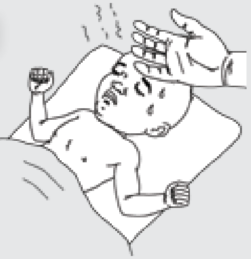

Fever ☐

For 7 days or more

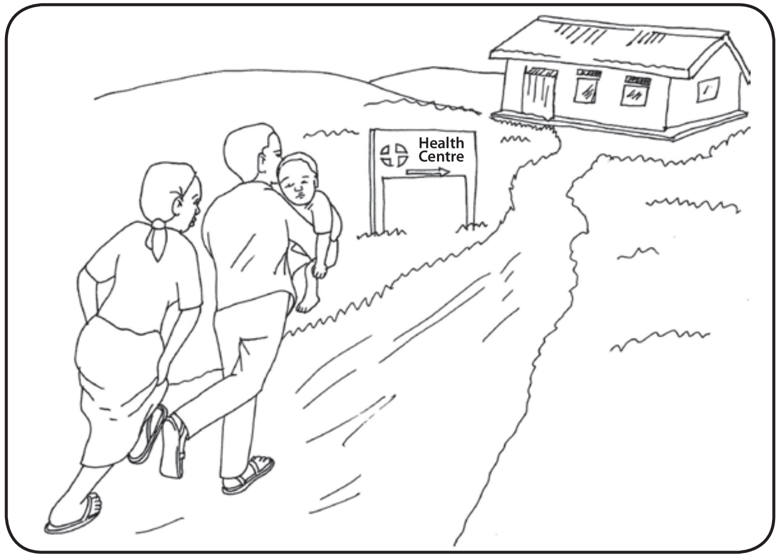

4a. PRE-REFERRAL TREATMENT

If the child has any of the danger signs below, give the following treatment:

Diarrhoea

For 14 days or more, or with blood in the stool

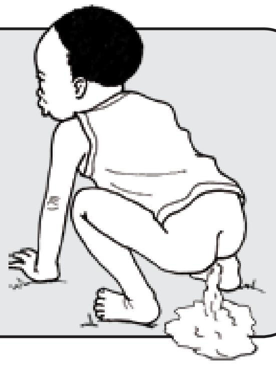

- ☐ Begin giving the child ORS before you refer. Advise caregiver to continue giving ORS to the child on the way to the health centre.

Chest In-drawing or Fast Breathing

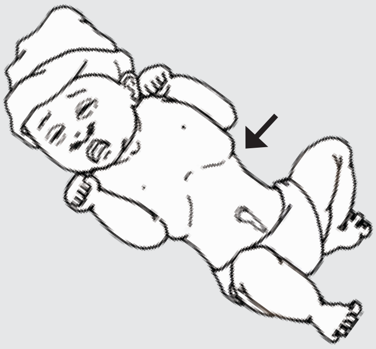

With a Danger Sign

- ☐ Give first dose of Amoxicillin before you refer.

2 - 11 months: 2 tabs from RED PACK

1 - 5 years: 3 tabs from GREEN PACK

Fever

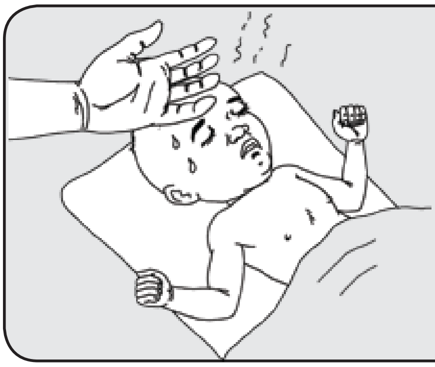

For 7 days or more

- ☐ Give first dose oral Anti-Malarial ACT.

4 months - 2 years: 1 tab from YELLOW PACK

3 - 5 years: 2 tabs from BLUE PACK

Fever and a General Danger Sign

- ☐ Give Rectal Artesunate.

4 - 11 months: 1 cap

1 - 3 years: 2 caps

4 - 5 years: 4 caps

- ☐ Help caregiver give dose.

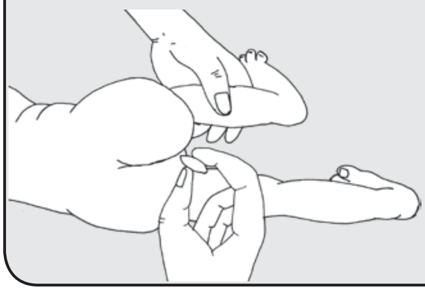

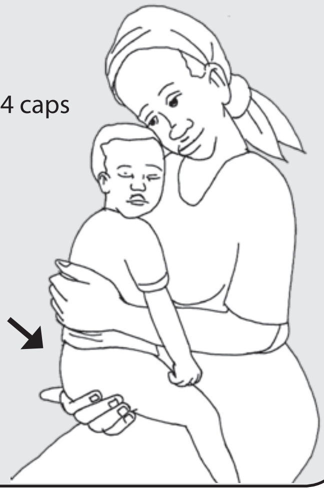

Newborn

With a Danger Sign

- ☐ **REFER**

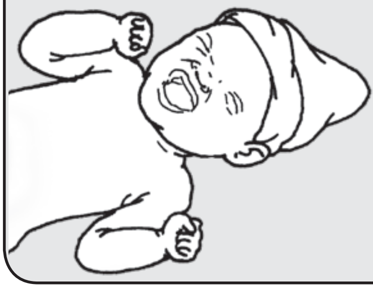

4b. TREAT AND ADVISE

If the child has any of the symptoms below with NO danger signs, give the following treatment:

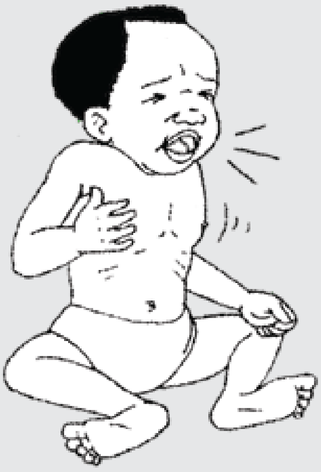

Cough

With fast breathing for less than 21 days

- ☐ Give oral antibiotic – Amoxicillin.
- ☐ Advise caregiver to give:

2 - 11 months: 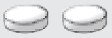 2 tabs twice a day for 5 days from RED PACK

1 - 5 years: 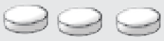 3 tabs twice a day for 5 days from GREEN PACK

- ☐ Help caregiver give first dose now.

Diarrhoea

For less than 14 days, and without blood in stool

- ☐ **Give ORS.** Help caregiver give child ORS solution in front of you until the child is no longer thirsty.
- ☐ **Give caregiver 2 ORS packets to take home.** 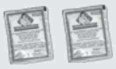 Advise caregiver to give as much as child wants, but at least ½ cup ORS solution after each loose stool.
- ☐ **Give zinc supplement.** Give 1 dose daily for 10 days.

2 to 6 months: 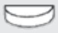 ½ a tab once a day for 10 days

6 months to 5 years: 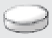 1 tab once a day for 10 days

- ☐ Help caregiver give first dose now.

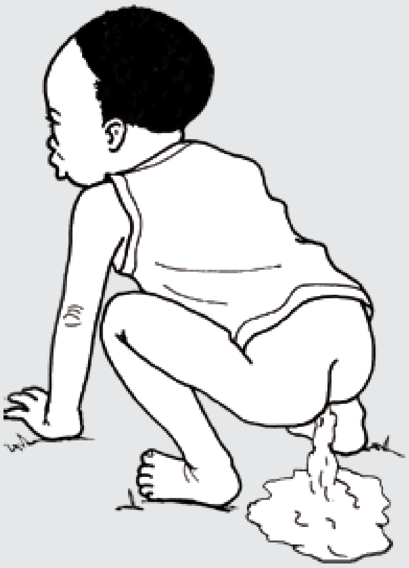

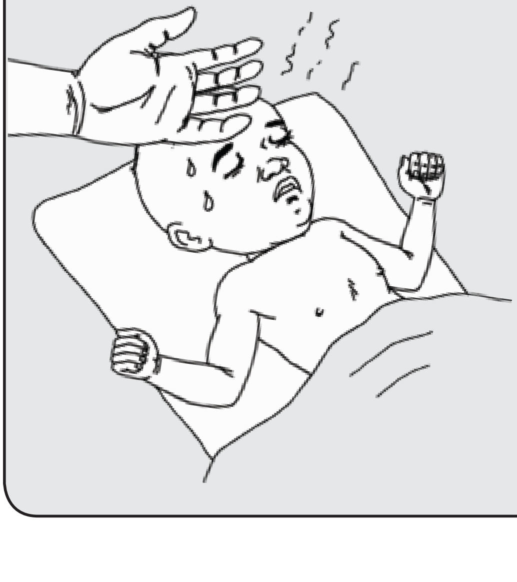

Fever

Less than 7 days

- ☐ Give oral Anti-Malarial ACT.

4 months to 2 years: 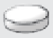 1 tab twice a day for 3 days from YELLOW PACK

3 years to 5 years: 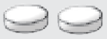 2 tab twice a day for 3 days from BLUE PACK

- ☐ Help caregiver give first dose now.
- ☐ Advise caregiver on use of a bed net (LLIN).

5. ADVICE FOR ALL CHILDREN TREATED AT HOME

Give more fluids and continue feeding

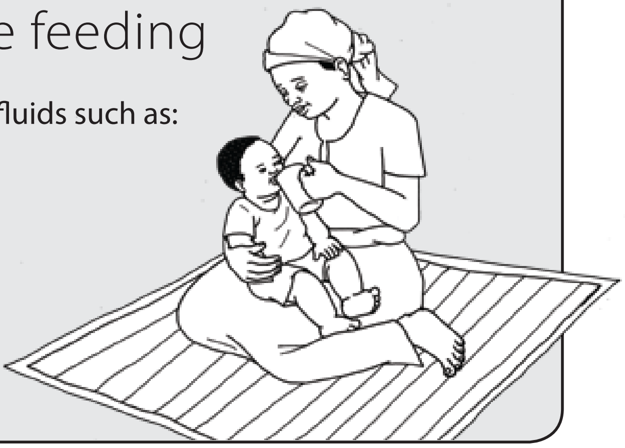

- ☐ Advise caregiver to give plenty of homemade fluids such as:
    - Clean water
    - Soup
    - Yoghurt drinks
    - Light porridge
    - ORS

Go to the health facility if...

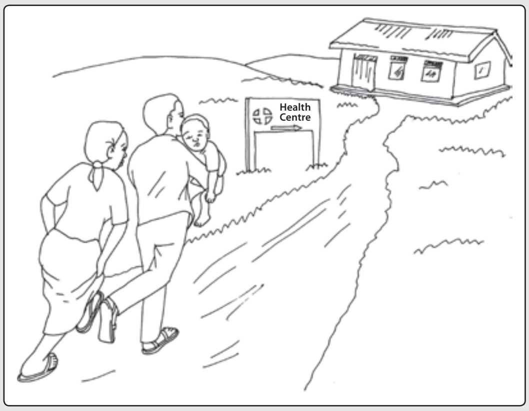

- ☐ Advise to go to the health facility if the child:
    - Cannot drink or feed
    - Has blood in stool
    - Becomes sicker
    - Develops any other danger sign
    - Has not improved in 2 days

Sleep under a net

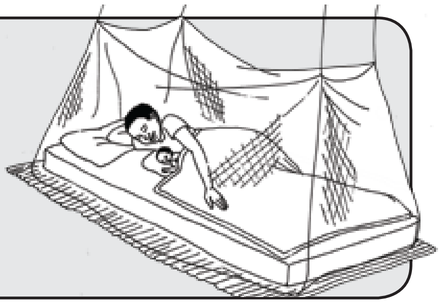

- ☐ Advise caregiver to be sure the child sleeps under a mosquito net.

Follow up child in 3 days

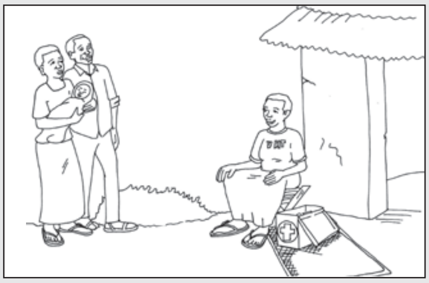

- ☐ Decide if you (the VHT member) will go to the child or if the caregiver will bring the child to you.

6. ROUTINE CARE FOR THE NEWBORN

Keeping the baby warm

Advise the caregiver on the following:

- ☐ Wrap the baby in warm, dry clothes, including the head and feet.
- ☐ Put the baby in skin-to-skin contact with the mother, as shown in the picture.
- ☐ Delay the baby's first bath until after 24 hours.

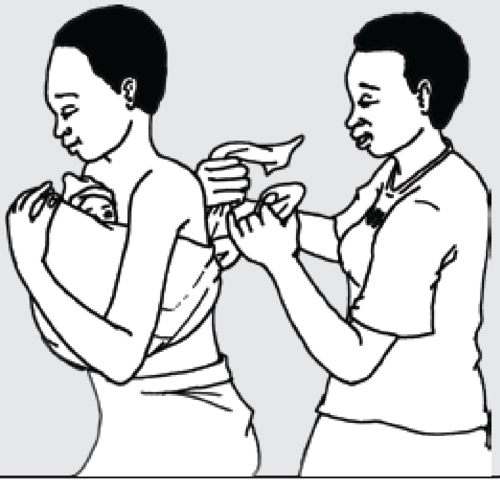

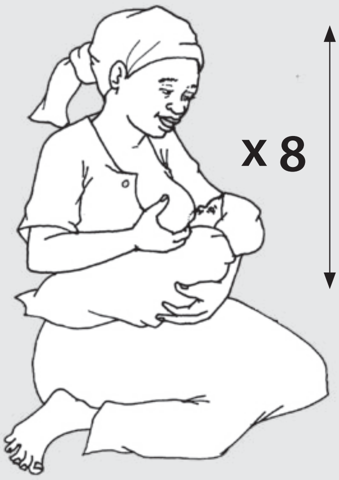

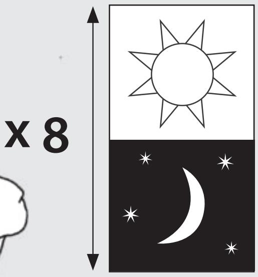

Breastfeeding exclusively

Advise the mother on the following:

- ☐ Start breastfeeding immediately after birth.
- ☐ Feed the baby on only breast milk, on demand, at least 8 times a day.
- ☐ Ensure the baby is well positioned and attached on the mother's breast.

Skin and cord care

Advise the caregiver on the following:

- ☐ Wash hands before handling the baby.

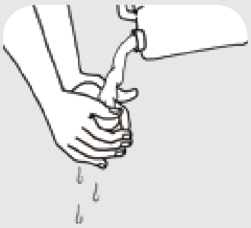

- ☐ Do not apply anything on the cord. Leave the cord dry and open.

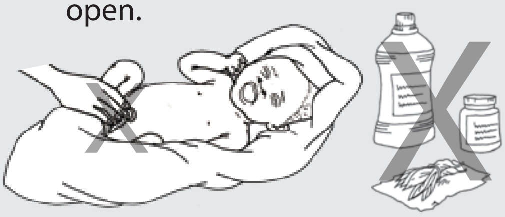

- ☐ Bathe the baby with clean soap and water.

THE REPUBLIC OF UGANDA
Ministry of Health

VHT
VILLAGE HEALTH TEAM

Designed and illustrated by **Mango Tree**
www.mangotreeuganda.org
